# Supervisor support and engineering identity among engineering postgraduates: engineering self-efficacy as a mediator and psychological capital as a moderator

**DOI:** 10.3389/fpsyg.2026.1816422

**Published:** 2026-05-14

**Authors:** Weiwei Li, Liangting Jia, Jinfeng Lu, Liangyu Cao

**Affiliations:** 1 Faculty of Education, East China Normal University, Shanghai, China; 2 Office of Development Planning, Shanghai Customs University, Shanghai, China; 3 School of Education Science and Technology, Nanjing University of Posts and Telecommunications, Nanjing, China; 4 Graduate School, Northwestern Polytechnical University, Xi’an, China

**Keywords:** engineering identity, engineering postgraduates, engineering self-efficacy, psychological capital, supervisor support

## Abstract

**Introduction:**

In contemporary society, engineering postgraduates face substantial academic and career-related pressures. Some students may choose to leave the engineering field during their study or employment stage. Notably, engineering postgraduates tend to report relatively low levels of engineering identity, a condition that may be linked to an increased risk of engineering talent attrition, warranting closer scholarly scrutiny. During postgraduate training, supervisors play a pivotal role, and the support they provide constitutes a critical resource for addressing this challenge. Consequently, it is crucial to systematically examine the relationship between supervisor support and engineering postgraduates’ engineering identity.

**Method:**

This study utilized a cross-sectional online survey design to collect data from 869 engineering postgraduates in China. Established and validated measurement instruments were employed to assess supervisor support, engineering self-efficacy, psychological capital, and engineering identity. A mediation and moderation model was tested to examine the relationship between supervisor support and engineering identity, the mediating role of engineering self-efficacy, and the moderating effect of psychological capital on the direct path.

**Results:**

The findings revealed that supervisor support was positively associated with engineering postgraduates’ engineering identity. Engineering self-efficacy mediated this relationship. Furthermore, psychological capital moderated the association between supervisor support and engineering identity, such that the positive relationship was stronger among postgraduates with higher psychological capital.

**Discussion:**

These results emphasize the significance of adopting effective supervisor support strategies and cultivating engineering postgraduates’ engineering self-efficacy and psychological capital, factors that are positively associated with engineering identity among Chinese engineering postgraduates.

## 1 Introduction

Amid the rapid advancement of the industrial revolution and ongoing technological transformation, engineers have emerged as a crucial driving force of national innovation, social progress, and economic growth worldwide. As a primary pipeline for future engineers, engineering postgraduates are widely regarded as a critical component of national strategic talent systems. However, many countries are generally facing the severe challenge of engineering talent loss, as many engineering graduates are not engaged in engineering or related professions (Striolo et al., 2021; Liquete et al., 2025). Consequently, how to effectively retain potential engineering talent has become an urgent practical priority for countries worldwide. Prior research indicates that students’ intentions to pursue engineering careers and their development into professional engineers are positively associated with stronger engineering identity, while weaker engineering identity is linked to a higher likelihood of leaving the field (Hughes et al., 2021). However, empirical study suggests that the engineering identity of engineering postgraduates remains at a relatively low level. Large-scale surveys have shown that engineering postgraduates, particularly those from Asian backgrounds and female students, report comparatively weaker engineering identity (Bahnson et al., 2021). Against this background, systematically examining the mechanisms underlying engineering identity and identifying feasible pathways for its enhancement have become pressing issues in higher engineering education.

Research on the formation of engineering postgraduates’ identity is still at an early stage, and the relevant literature remains limited (Rodriguez et al., 2018). Prior studies have predominantly concentrated on the external support environment and individual internal factors. Research on the external environment has examined factors such as laboratory composition (Crede and Borrego, 2012; Perkins et al., 2018), study experience (Bahnson et al., 2019), academic relations along with degree program study time (Bahnson et al., 2021), and interpersonal relationships (Perkins et al., 2020), all of which are related to engineering postgraduates’ identity. In contrast, studies on individual internal factors have explored the roles of research interest (Bahnson et al., 2023), engineering belonging (Li et al., 2026), and personal ability (Choe and Borrego, 2020). Generally, most studies exploring the relationship between external environments and engineering identity focus on isolated dimensions, while those addressing the mediating role of individual psychological factors remain relatively sparse. Greater integration might be achieved in identifying key external environmental variables and exploring how internal individual factors interact with them in relation to engineering identity.

In the Chinese postgraduate training system, supervisors are the most powerful actors in master’s development (Wang et al., 2022a). Consequently, supervisor support is recognized as a critical environmental factor closely linked to postgraduates’ academic and professional development (Zhang C. et al., 2022; Zhang S. et al., 2022). High-quality supervisor support can provide engineering postgraduates with both instrumental and psychological resources that facilitate professional development and may be associated with growth in research competence, confidence, and professional identity (Gehr et al., 2023). At present, the research on supervisor support has primarily examined its associations with postgraduates’ academic productivity (Angrist and Diederichs, 2024; Lunsford, 2012), innovative capacity (Zhang C. et al., 2022; Zhang S. et al., 2022), and career development (Martins and Faciola, 2025). However, the studies have rarely involved the deep psychological construction of engineering identity. The development of engineering postgraduates’ identity is considered to be related to both environmental factors and internal personal characteristics (Eliot and Turns, 2011). Therefore, incorporating individual characteristics into analyses of the relationship between external environments and engineering identity can provide a more comprehensive basis for designing interventions aimed at supporting postgraduates’ engineering identity. Engineering self-efficacy and psychological capital represent two key individual-level psychological resources. Engineering self-efficacy is the extent to which individuals are confident in their capability to proficiently execute engineering projects (Phan et al., 2023). Postgraduates with higher engineering self-efficacy are more inclined to proactively address challenging problems in engineering tasks. This active engagement in continuous practice, in turn, is linked to stronger reported levels of professional belonging and recognition of value among postgraduates. Conversely, these patterns are less evident among engineering postgraduates with lower engineering self-efficacy (Sperling et al., 2024). Psychological capital is a favorable psychological state that individuals demonstrate throughout their developmental process (Luthans et al., 2006a,b). It has also been identified as an important factor associated with engineering postgraduates’ professional identity (Gong et al., 2025; Deng, 2024). Therefore, incorporating individual characteristics closely associated with engineering identity, like engineering self-efficacy and psychological capital, is of great significance for clarifying the specific path and internal mechanisms underlying the relationship between supervisor support and engineering postgraduates’ engineering identity.

In summary, this study focuses on engineering postgraduates and draws upon Person-Environment Interaction Theory (PEIT) and Social Cognitive Career Theory (SCCT) to examine the relationship between supervisor support and engineering identity. By examining the mediating role of engineering self-efficacy and the moderating role of psychological capital, this study aims to provide evidence-based implications for enhancing supervisor support practices, deepening the understanding of factors associated with engineering postgraduates’ identity in China, and ultimately contributing to the development of a high-quality and sustainable engineering workforce.

## 2 Literature review and hypothesis

### 2.1 Theoretical framework

PEIT posits that professional identity is dynamically linked to ongoing interactions between external environmental factors and individuals’ internal characteristics—a relationship reflecting its dynamic construction process (Guan et al., 2021). Within the structure, supervisor support serves as a fundamental environmental resource associated with postgraduates’ recognition of the engineering role, whereas psychological capital, as an internal personal resource (Luthans et al., 2006a,b; Luthans and Youssef-Morgan, 2017), is related to how environmental influences are interpreted and translated into identity development. Although this theory establishes the basic logic of individual-environment interaction, it does not specify how external environmental factors correlate with identity outcomes via internal cognitive processes. SCCT helps address this limitation. Specifically, the theory identifies self-efficacy as the primary cognitive mechanism connecting environmental support to individual career development (Flores et al., 2021). In engineering education, engineering self-efficacy serves as the domain-specific manifestation of this mechanism. Research shows that engineering self-efficacy primarily comes from mastery experiences, vicarious learning, verbal persuasion, and emotional arousal, all of which are closely associated with supervisors’ academic guidance, emotional encouragement, and autonomy support (Amador-Campos et al., 2023). Therefore, SCCT offers a more specific account of the mediating pathway through which supervisor support is associated with engineering identity.

It is important to distinguish engineering self-efficacy from psychological capital in this model with respect to their theoretical level and functional role. Engineering self-efficacy is a domain-specific cognitive belief that functions as a mediator, illustrating the associative pathway through which supervisor support is linked to engineering identity. By contrast, psychological capital is a cross-situational positive psychological resource that functions as a moderator, indicating the conditions under which the association between supervisor support and identity outcomes is stronger. According to PEIT, the primary function of internal personal resources is related to how people’s perceptions and reactions to the external environment, rather than being associated with changes in the cognitive transformation process itself (Umbra and Fasbender, 2025). Thus, psychological capital, as a higher-order personal resource, primarily moderates the direct “environment → outcome” relationship. Engineering self-efficacy, as a cognitive belief, represents the “environment → cognition → outcome” mediational pathway. Functionally, the two complement each other as “cognitive dimensions” and “boundary conditions,” offering a comprehensive account of the connection between supervisor support and engineering identity.

**Figure 1:**
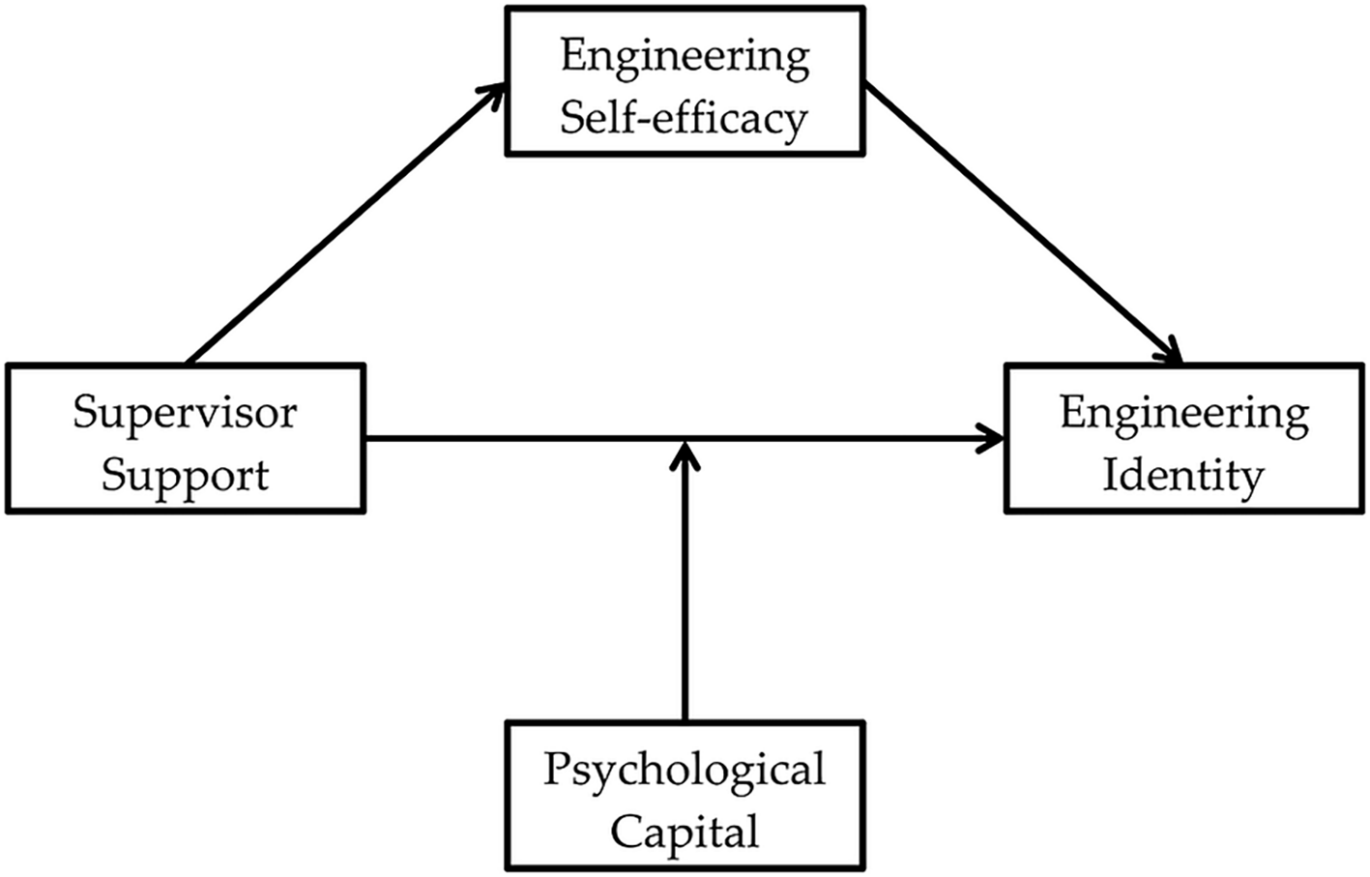
Theoretical model.

Based on the complementarity of these two theoretical perspectives, this study constructs an integrated mediation and moderation model (see Figure 1). Engineering identity is positioned as the core outcome variable: consistent with PEIT, it is associated with the interaction between the individual and environment as ultimate aspect; according to SCCT, it is linked to the career development process as a terminal dimension, reflecting the state in which an individual internalizes their professional role into their self-concept. Engineering self-efficacy is specified as the central mediating variable. This choice is supported not only by SCCT but also by empirical evidence identifying it as one of the strongest associates of engineering identity (Wei et al., 2025). Psychological capital is positioned as the moderating variable, reflecting the correlation between individuals’ internal psychological resources and the strength of the association between external environmental support and engineering identity. This integrated framework not only clarifies the overarching logic of “environment-individual interaction” but also specifies the sequential pathway of “environment → cognition → identity,” and offers a coherent theoretical foundation for the formulation of subsequent hypotheses.

### 2.2 Supervisor support and engineering identity

Supervisor support denotes the supportive behaviors through which supervisors are intended to facilitate postgraduates’ academic advancement and personal development. Supervisor support is typically conceptualized as a multidimensional construct. Overall et al. (2011) emphasized academic, autonomy, and personal support. In the Chinese context, He and Zhu (2023) proposed a four-dimensional framework encompassing academic, interpersonal, autonomy, and moral support, whereas Ma et al. (2023) summarized supervisor support into instrumental and emotional support. Although there are differences in the definition of supervisor support from different theoretical perspectives and cultural backgrounds, existing studies generally regard academic, emotional, and autonomy support as their core components. In this study, we adopt this classification, grouping supervisor support into these three categories. Academic support focuses on providing substantive support for the academic research of engineering postgraduates, covering the necessary knowledge transfer and resource support; emotional support focuses on guiding engineering postgraduates to have positive emotions, and the main support methods are approval, encouragement, motivation, etc.; autonomy support mainly involves respecting engineering postgraduates’ views, encouraging open expression, and providing opportunities for independent decision-making.

The concept of “engineering identity” comes from Gee (2000), who defined identity as “being regarded as a specific type of individual,” which represents an external classification perspective. Potvin and Hazari (2013) noted that this externally oriented framework has limitations. It fails to fully capture individuals’ internal psychological experiences. Subsequent studies emphasized incorporating subjective dimensions such as self-perception and self-positioning into the conceptualization. Research on engineering identity has developed three orientations. Firstly, the internal perspective, which defines engineering identity in terms of individuals’ self-perceptions of their engineering roles and competence (Jones et al., 2014). Secondly, the externally oriented perspective defines engineering identity as the sensation of belonging to the engineering community experienced by engineering postgraduates (Major and Kirn, 2017). Thirdly, the integrative perspective, which combines internal and external orientations, advocates that engineering identity involves both individuals’ internal self-positioning and recognition by others (Perkins et al., 2017). This study adopts the conceptual model proposed by Godwin and Kirn (2020), which characterizes engineering identity as the degree to which engineering postgraduates regard themselves as capable and impactful professionals within the engineering domain. This model comprises three dimensions: interest, recognition, and performance/competence. The model aligns closely with the educational context of Chinese engineering postgraduates, and its measurement instruments have been empirically validated (Lockhart et al., 2025), demonstrating strong reliability and validity. It provides an appropriate framework for this study.

SCCT posits that the establishment and evolution of engineering identity among engineering postgraduates are closely related to individual cognitive elements and external environmental factors (Lent et al., 1994). As an important part of external factors, supervisors are the “guides” on the development path of engineering postgraduates. Their roles and behaviors are considered intrinsically linked to the formation of engineering postgraduates’ professional identity (Gruber et al., 2023). Numerous studies have confirmed the correlation between supervisor support and the development of postgraduates’ engineering identity. For instance, Chemers et al. (2011) discovered that effective supervision is associated with stronger professional identity and greater commitment to scientific careers among engineering students. A large-scale survey of 1754 engineering postgraduates showed that favorable supervisor interactions correlate with elevated engineering identity levels (Perkins et al., 2020). Bahnson et al. (2021) reported variations in engineering identity at both personal and disciplinary levels and found that high-quality supervisor support correlated positively with students’ identification with the engineer role. In summary, the support supplied by the supervisor is positively associated with postgraduates’ engineering identity. Consequently, the research hypotheses are proposed:

*H1*: Supervisor support is positively associated with engineering postgraduates’ engineering identity.

### 2.3 Engineering self-efficacy as the mediator

The concept of self-efficacy was first put forward by Bandura (1977). As research on self-efficacy expanded, the construct was widely applied to domains such as education, career development, and organizational behavior (Shi et al., 2025; García-Álvarez et al., 2024; Betz and Hackett, 1981; Rizzo, 2023). Engineering self-efficacy refers to the specialized use of self-efficacy in the realm of engineering. Engineering self-efficacy is the overarching belief among engineering postgraduates in their ability to execute engineering-related projects effectively. As discussed in the theoretical framework, supervisors’ academic guidance, emotional encouragement, and autonomy support serve as critical sources associated with the development of engineering postgraduates’ engineering self-efficacy. Existing studies have shown a positive correlation between supervisor support and the engineering self-efficacy of engineering postgraduates. Systematic academic guidance from supervisors may enable postgraduates to accumulate mastery experiences in addressing complex engineering challenges, seen as a fundamental source of self-efficacy beliefs (Paglis et al., 2006; Rivera et al., 2023). Additionally, supervisors’ emotional care and encouragement may help postgraduates maintain a positive understanding of their own abilities and reduce self-doubt, which may be associated with the consolidation of their engineering self-efficacy (Mantai, 2019). By providing autonomy in research topic selection and methodological decision-making, supervisors may further allow postgraduates to experience successful independent exploration. This sense of control may be further linked to their stronger conviction in accomplishing engineering tasks (Devos et al., 2015). Consequently, supervisor support is anticipated to have a favorable association with engineering self-efficacy in engineering postgraduates.

Social cognitive theory posits that self-efficacy, as an innate cognitive and motivational element, correlates with individuals’ cognitive processes, emotional experiences, and behavioral decisions. It is also linked to the internalization of professional roles through behavioral engagement and sustained participation (Bandura, 1991). In engineering education, postgraduates with elevated engineering self-efficacy are significantly more inclined to take part actively in engineering practices and professional groups. Such sustained engagement and sense of belonging are associated with their gradual internalization of the engineer role into their self-concept, which is related to the formation of a stable engineering identity. This internal belief system endures from university training into early career, shaping their sense of self as an engineer (Mahajan, 2024). Empirical research has consistently demonstrated a significant relationship between engineering self-efficacy and engineering identity. Through a systematic review, Rodriguez et al. (2018) identified engineering self-efficacy as a key psychological factor underlying professional identity development among engineering postgraduates. Similarly, Chemers et al. (2011) discovered that when engineering postgraduates have a high sense of engineering self-efficacy, they are more likely to develop strong beliefs regarding professional engineering roles. These beliefs are gradually internalized into their “professional self-concept” and are accepted and recognized by individuals. Therefore, we put forward the following assumptions:

*H2*: Engineering self-efficacy mediates the relationship between supervisor support and the engineering identity of engineering postgraduates.

### 2.4 Psychological capital as the moderator

Psychological capital denotes the affirmative psychological resources that individuals cultivate during their developmental stages, encompassing the four qualities of self-efficacy, hope, optimism, and resilience (Luthans and Youssef, 2004). In fact, psychological capital is not a psychological state or personality trait but a fusion of the two, manifested as a “state-like” positive psychological force that combines stability with malleability (Luthans and Youssef, 2007; Luthans et al., 2007). As a key personal resource, psychological capital is considered to play a core driving and buffer role in the process of individuals responding to the external environment and promoting the development of self-awareness (Han J. et al., 2022; Han X. et al., 2022). These functions are particularly relevant within the framework of engineering postgraduate development.

Positive supervisor support may be positively related to engineering postgraduates’ engineering identity. However, even under the condition that the perceived level of tutor support is similar, there are certain individual differences in the engineering identity of different engineering postgraduates. This suggests that engineering identity is linked to both external environmental influences and individual characteristics. Under similar external support conditions, the reason for the difference in engineering identity between individuals may be partly due to individual factors. Psychological capital might serve as a substantial moderating factor in the connection between supervisor support and engineering identity. Psychological Capital Theory suggests that postgraduates possessing enhanced psychological capital are more adept at perceiving, internalizing, and utilizing external resources, potentially transforming contextual support into opportunities for self-development (Li et al., 2023). This process may lead to positive psychological experiences. Positive psychological experiences are connected with a more enhanced professional and career identity. Burleson et al. (2021) discovered that individuals with greater psychological capital were better able to perceive the meaning and value of engineering learning experiences, which are positively associated with engineering identity. Postgraduates who perceive strong supervisor support tend to exhibit higher intrinsic motivation, greater self-efficacy, and stronger confidence in becoming engineers, all of which are positively associated with engineering identity. Conversely, individuals with poorer psychological capital may experience greater stress and diminished capacity to benefit from supervisor support, which may be associated with lower identification with their academic program and chosen profession (Hope et al., 2022). Zhang C. et al. (2022) and Zhang S. et al. (2022) further indicated that low psychological capital individuals are more vulnerable to emotional and cognitive exhaustion, which are associated with lower professional identity. Therefore, we propose the following hypothesis:

*H3*: Psychological capital moderates the relationship between supervisor support and engineering postgraduates’ engineering identity.

## 3 Methods

### 3.1 Participants

Data were collected through a questionnaire survey of engineering postgraduates in Chinese universities conducted in November and December 2025. The electronic questionnaire was distributed indirectly by the authors and was coordinated through graduate school administrators and other academic supervisors to engineering postgraduates through WeChat, QQ, and email. There were 964 responses in all. The respondents were excluded if their questionnaires were incomplete or indicated a straight-lined pattern. We also screened out questionnaires completed in less than 60 s. After these exclusions, 869 valid surveys were retained, resulting in an effective response rate of 90.15%. As suggested, the sample size was checked to make sure it was right for using Structural Equation Modelling (SEM). The minimum sample for SEM should be tenfold the quantity of observed variables (Kline, 1998). The questionnaire of this study has 47 items, meaning that the minimum sample size should be 470. Thus, the final sample meets the statistical guidelines for an SEM. The demographic details of the participants are shown in Table 1.

**Table 1:** Engineering postgraduates demographics (*N* = 869).

Variable		Frequency (*n*)	Percentage (%)
Gender	Male	457	52.59%
	Female	412	47.41%
Education level	Master student	469	53.97%
	Doctoral student	400	46.03%
Type of university	Other universities	415	47.76%
	Double First-Class universities	454	52.24%
Engineering practice experience	Non-practice-experienced	477	54.89%
	Practice-experienced	392	45.11%

### 3.2 Scale revision and validation

To ensure cultural relevance, linguistic precision, and psychometric validity, we undertook a systematic review of the measures. This started with a literature search to ascertain if there were already scales for each of the conceptual constructs, followed by a round of independent forward and back translation of the instruments to make them linguistically equivalent for the study; two rounds of content validity were assessed, in the first five engineering experts rating the theoretical relevance and contextual fit of items, with some initial revisions made; this was followed by a round of review with 10 engineering postgraduates focusing on semantic clarity and contextual fit. In October 2025, 134 engineering postgraduates participated in a pilot study. Reliability analysis showed Cronbach’s *α* values for the four dimensions above 0.79, indicating acceptable internal consistency. Also, the Kaiser–Meyer–Olkin value was 0.801; Bartlett’s test yielded a significant result (χ^2^ = 2912.955, *p* < 0.001), demonstrating the data’s appropriateness for factor analysis. Exploratory factor analysis was performed using the criterion of primary factor loading greater than 0.50 and no substantial cross-loadings. Two items were deleted because of low loading. The final solution showed the four-factor structure was clear and accounted for 60.86% of the total variance. All items had factor loadings ranging from 0.52 to 0.75, providing evidence of their empirical validity.

### 3.3 Measurement

#### 3.3.1 Supervisor support

Supervisor support was assessed using a scale adapted from Overall et al. (2011) and modified to align with the context of engineering postgraduate advisement. The 12-item instrument measures three dimensions. Responses were recorded on a 5-point Likert scale. Higher scores represent stronger perceived supervisor support. The scale demonstrated strong internal consistency in this sample (Cronbach’s *α* = 0.888). A confirmatory factor analysis (CFA) supported the hypothesized three-factor structure, with fit indices indicating acceptable model fit: χ^2^/df = 1.429, RMSEA = 0.022, SRMR = 0.020, CFI = 0.979, TLI = 0.983.

#### 3.3.2 Engineering self-efficacy

Engineering self-efficacy was measured using six items adapted from Mamaril et al. (2016) and further refined in light of the postgraduate engineering education context in China. Items were rated on the same 5-point Likert scale, where higher scores reflect domain-specific efficacy beliefs. The scale showed acceptable internal consistency (Cronbach’s *α* = 0.803). CFA supported the unidimensional structure, with the model fit being good: χ^2^/df = 1.756, RMSEA = 0.042, SRMR = 0.025, CFI = 0.973, TLI = 0.970.

#### 3.3.3 Psychological capital

Psychological capital was evaluated with a 16-item scale integrating items from Luthans et al. (2006a,b) and Wen et al. (2009), adapted for graduate academic settings. All items were rated on a 5-point Likert response scale, with higher average scores indicating greater psychological capital. The composite scale exhibited excellent reliability (Cronbach’s α = 0.895). A correlated four-factor CFA model yielded excellent fit: χ^2^/df = 1.313, RMSEA = 0.011, SRMR = 0.018, CFI = 0.986, TLI = 0.985.

#### 3.3.4 Engineering identity

Engineering identity was measured using 13 items adapted from Godwin and Kirn (2020) and Choe and Borrego (2019), tailored to the context of Chinese engineering postgraduate education. Responses were recorded on a 5-point Likert scale with higher scores indicating stronger engineering identity. The scale’s internal consistency was high (Cronbach’s α = 0.894). CFA validated the three-factor structure, with excellent fit indices: χ^2^/df = 1.139, RMSEA = 0.013, SRMR = 0.017, CFI = 0.989, TLI = 0.987.

### 3.4 Discriminant validity

On the basis of verifying the structural validity of each scale, this study further adopts the Fornell-Larcker criterion (Fornell and Larcker, 1981) and heterotrait-monotrait ratio of correlations (Henseler et al., 2015) to examine discriminant validity among the four core constructs: supervisor support, engineering self-efficacy, psychological capital, and engineering identity. Table 2 illustrates that the square root of the average variance extraction (AVE) for each idea is 0.763, 0.737, 0.716, and 0.754, respectively, all exceeding the correlation coefficient between each concept and any other concept. Notably, the correlation between engineering self-efficacy and psychological capital was 0.210, which was lower than the square root of the AVE for each construct, indicating satisfactory discriminant validity. The heterotrait-monotrait ratios were also computed; all values ranged from 0.18 to 0.64, falling below the conservative threshold of 0.85, with the heterotrait-monotrait value between engineering self-efficacy and psychological capital being 0.32. Collectively, these findings indicate that the four core constructs in this study exhibit adequate discriminant validity.

**Table 2:** Discriminant validity.

Variable	1	2	3	4	AVE
1. Supervisor support	**0.763**				0.582
2. Engineering self-efficacy	0.429	**0.737**			0.543
3. Psychological capital	0.219	0.210	**0.716**		0.512
4. Engineering identity	0.396	0.555	0.153	**0.754**	0.568

Diagonal values (bold) represent the square roots of the average variance extracted (AVE). Off-diagonal values represent inter-construct correlations. All correlations are significant at *p* < 0.01.

### 3.5 Statistical analysis

This study used SPSS and Amos for data analysis. First, measurement model validation was conducted using Amos 26.0. CFA assessed the construct validity and internal consistency of the multi-dimensional scales. Second, SPSS 27.0 was used for preliminary analyses. Descriptive statistics summarized the central tendency and distribution of study variables. Subsequently, correlation analysis was conducted to examine the bivariate associations among these constructs, providing an initial overview of their interrelationships. Hypothesis testing was performed using PROCESS v4.0 for SPSS, which is explicitly tailored for evaluating mediation and moderation models. Given that our model involves both mediating and moderating mechanisms, PROCESS was deemed appropriate for directly estimating the hypothesized relationships. Specifically, Model 4 was employed to test the mediating role of engineering self-efficacy, and Model 5 was employed to test the moderating role of psychological capital (Hayes, 2013). In the moderation analysis, psychological capital was specified as a moderator of the direct path between supervisor support and engineering identity.

### 3.6 Ethical considerations

This study was approved by East China Normal University. Written informed consent was obtained from all participants. No personally identifiable information was collected, and all data were used solely for academic research purposes.

## 4 Results

### 4.1 Common method bias test

This study used two methods to evaluate common method bias (Chin et al., 2012). Harman test results showed the first unrotated factor accounted for 21.22% of total variance, below the 40% critical standard. Two CFA models were compared to examine method bias further: a baseline model (M1) and a model with a latent method factor (M2). Model fit index differences between M1 and M2 were small (ΔCFI = 0.021, ΔRMSEA = 0.008, ΔSRMR = 0.004). These modifications remained below the suggested cutoff of 0.03, indicating that the common method factor did not significantly enhance model fit. These results show that common method bias, while not entirely ruled out, does not significantly threaten the study’s conclusions’ validity.

### 4.2 Descriptive statistics and correlation analysis

Table 3 presents the descriptive statistics and intercorrelations of the four central variables. The mean scores indicate that supervisor support (*M* = 2.69, *SD* = 0.72), engineering self-efficacy (*M* = 2.75, *SD* = 0.77), psychological capital (*M* = 2.67, *SD* = 0.68), and engineering postgraduates’ engineering identity (*M* = 2.66, *SD* = 0.72) are in the lower-middle range. Pearson correlation analysis revealed that supervisor support was significantly and positively associated with engineering self-efficacy (*r* = 0.429, *p* < 0.01), psychological capital (*r* = 0.219, *p* < 0.01), and engineering identity (*r* = 0.396, *p* < 0.01). Furthermore, engineering self-efficacy was also positively associated with psychological capital (*r* = 0.210, *p* < 0.05) and engineering identity (*r* = 0.555, *p* < 0.01). Psychological capital showed a modest but significant positive association with engineering identity (*r* = 0.153, *p* < 0.01). These findings offer initial evidence in favour of the proposed connections.

**Table 3:** Descriptive statistics and correlation coefficients (*N* = 869).

Variable	M	SD	1	2	3	4
1. Supervisor support	2.69	0.72	1			
2. Engineering self-efficacy	2.75	0.77	0.429**	1		
3. Psychological capital	2.67	0.68	0.219**	0.210**	1	
4. Engineering identity	2.66	0.72	0.396**	0.555**	0.153**	1

**p < 0.01.

### 4.3 Testing of the mediation model

To investigate the mediating effect of engineering self-efficacy between supervisor support and engineering identity among engineering postgraduates, PROCESS Model 4 was employed. Gender, degree type, university type, academic performance ranking, and practical experience were included as control variables. Using the bias-corrected percentile bootstrap method, 5,000 samples were repeatedly drawn from the original data to calculate the 95% bias-corrected confidence interval (CI). A mediation effect was considered significant when the CI did not include zero. As shown in Table 4, the overall model explained 37.1% of the variance in engineering identity (*R*^2^ = 0.371) and was statistically significant (*F* = 84.659, *p* < 0.001). Model 1 revealed a significant positive association between supervisor support and engineering identity (*b* = 0.40, *t* = 13.45, *p* < 0.001), supporting H1. Model 2 shows that supervisor support is significantly and positively associated with engineering self-efficacy (*b* = 0.46, *t* = 14.29, *p* < 0.001). In Model 3, engineering self-efficacy was significantly positively associated with engineering identity (*b* = 0.41, *t* = 14.43, *p* < 0.001). Concurrently, the direct association between supervisor support and engineering identity remained significant (*b* = 0.21, *t* = 7.16, *p* < 0.001).

**Table 4:** Testing the mediating effect of engineering self-efficacy.

Variable	Engineering identity (Model 1)		Engineering self-efficacy (Model 2)		Engineering identity (Model 3)	
	b	t	b	t	b	t
Gender	−0.09	−2.01*	−0.03	−0.71	−0.07	−1.89
Degree level	−0.30	−6.84***	−0.24	−4.98	−0.21	−5.09***
School type	−0.12	−2.78**	−0.02	−0.50	−0.11	−2.85**
Engineering practice experience	0.02	0.56	0.01	0.24	0.02	0.51
Supervisor support	0.40	13.45***	0.46	14.29***	0.21	7.16***
Engineering self-efficacy					0.41	14.43***
*R* ^2^	0.219		0.210		0.371	
F	48.319***		45.821***		84.659***	

*p < 0.05; **p < 0.01; ***p < 0.001.

Table 5 shows the mediation analysis results in detail. The Bootstrap test results revealed that the direct association between supervisor support and engineering identity [95% CI = (0.15,0.28)] and the indirect association via engineering self-efficacy [95% CI = (0.15,0.23)] were both significant. The direct effect value (0.21) and the indirect effect value (0.19) accounted for 53.13% and 46.87% of the total effect (0.40), respectively. The results indicate that engineering self-efficacy partially mediated the connection between supervisor support and engineering identity, supporting H2.

**Table 5:** Total, direct and mediating effect decomposition table.

Path	Effect	Boot SE	Boot LLCI	Boot ULCI	Effect Rate
Total effect	0.40	0.03	0.34	0.46	
Direct effect	0.21	0.03	0.15	0.28	53.13%
Indirect effect	0.19	0.02	0.15	0.23	46.87%

### 4.4 Testing the moderating model

To test the hypothesized moderating function of psychological capital, Model 5 from Hayes’ PROCESS macro was employed. All analyses included gender, degree type, institutional classification, academic performance ranking, and practical experience as covariates. As presented in Tables 6, a significant interaction was observed between supervisor support and psychological capital in relation to engineering postgraduates’ engineering identity (*b* = 0.09, *t* = 2.23, *p* < 0.05). This result indicates that psychological capital moderates the direct relationship between supervisor support and engineering identity, such that the strength of this association varies with the level of psychological capital. In the moderation model, the main effect of supervisor support was non-significant (*b* = −0.02, *t* = −0.18, *p* > 0.05), whereas the main effect of psychological capital was significantly negative (*b* = −0.22, *t* = −2.03, *p* < 0.05).

**Table 6:** Testing the moderating effect of psychological capital.

Variable	Engineering self-efficacy		Engineering identity	
	b	t	b	t
Gender	−0.03	−0.71	−0.07	−1.89
Degree level	−0.24	−4.98***	−0.20	−5.02***
School type	−0.02	−0.50	−0.11	−2.83**
Engineering practice experience	0.01	0.24	0.02	0.53
Supervisor support	0.46	14.29***	−0.02	−0.18
Engineering self-efficacy			0.40	13.88***
Psychological capital			−0.22	−2.03*
Supervisor support × Psychological capital			0.09	2.23*
R^2^	0.210		0.375	
F	45.821***		64.375***	

*p < 0.05; **p < 0.01; ***p < 0.001.

**Figure 2:**
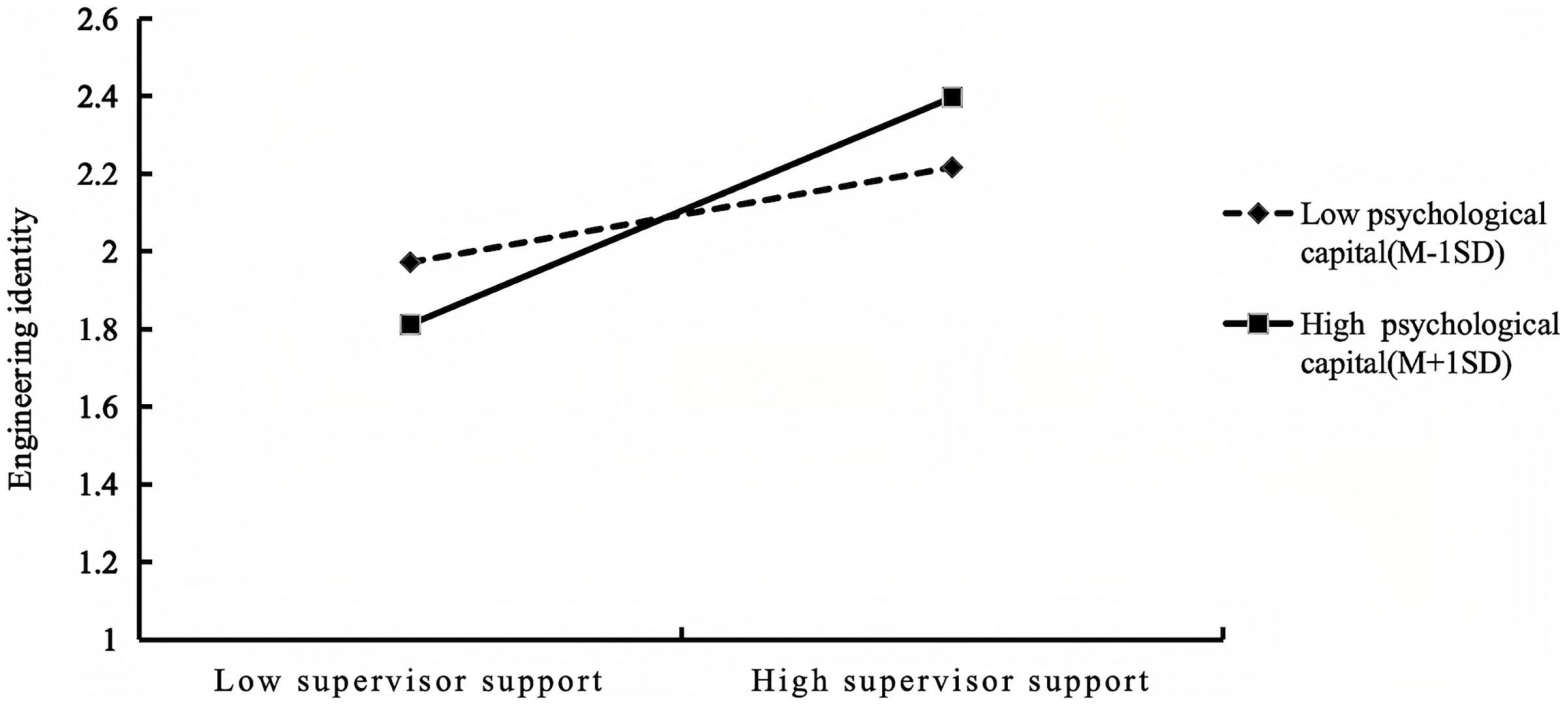
The moderating effect of psychological capital on the relationship between supervisor support and engineering identity. Simple slope plots are presented for low psychological capital (M−1 SD) and high psychological capital (M+1 SD).

To probe the nature of this interaction, simple slope analysis was conducted (see Table 7 and Figure 2). Following established practices, we examined the association between supervisor support and engineering identity at two levels of psychological capital: low psychological capital (M−1SD) and high psychological capital (M+1SD). For postgraduates with lower levels of psychological capital (M−1SD), supervisor support remained significantly and positively associated with engineering identity (simple slope = 0.150, *t* = 3.647, *p* < 0.001). For those with higher psychological capital (M+1SD), this positive association was substantially stronger (simple slope = 0.265, *t* = 6.912, *p* < 0.001). In other words, at higher levels of psychological capital, the positive relationship between supervisor support and engineering identity is more pronounced. These results suggest that psychological capital serves as a reinforcing factor, associated with a stronger positive association between supervisor support and engineering identity. As psychological capital was specified as a moderator of the direct path only, conditional indirect effects were not examined in this model. Thus, H3 was supported.

**Table 7:** Moderating effects at different levels of psychological capital.

Variable	Effect	Boot SE	Boot LLCI	Boot ULCI
Mean−1SD	0.150	0.041	0.069	0.231
Mean	0.207	0.030	0.148	0.267
Mean+1SD	0.265	0.038	0.190	0.340

## 5 Discussion

### 5.1 Supervisor support and engineering postgraduates’ engineering identity

The research found that supervisor support is positively associated with engineering identity among engineering postgraduates. Most prior studies have concentrated on supervisor support concerning research productivity, academic satisfaction, and career development, with limited attention to the intrinsic connection and underlying mechanisms between supervisor support and engineering identity. From the perspective of identity theory, engineering identity is not statically ascribed but dynamically constructed through the ongoing interaction between individuals and their environment. In the Chinese postgraduate training system, supervisors serve as critical guides; the academic, emotional, and autonomy support they provide may serve as key environmental resources for postgraduates to internalize the engineer role into self-concept (Martin et al., 2024). The core of identity construction lies in the integration of “practical participation” and “meaning-making” (Tomlinson and Jackson, 2019). Supportive supervisory practices create opportunities for legitimate peripheral participation—by engaging in authentic or near-authentic engineering tasks, postgraduates gradually develop belonging and commitment to the engineering profession (Atkins et al., 2020). This process helps explain why supervisor support is associated with engineering identity. Longitudinal studies have further demonstrated that developmentally appropriate support aligned with engineering competencies is associated with positive academic experiences and professional self-concepts (Estrada et al., 2018; Byars-Winston et al., 2015). Although causal inferences cannot be drawn from cross-sectional data, this study provides empirical evidence from Chinese engineering postgraduates that corroborates the positive association between supervisor support and engineering identity.

### 5.2 The mediating role of engineering self-efficacy

The current research discovered that supervisor support is associated with engineering identity both directly and indirectly through engineering self-efficacy. This finding aligns closely with SCCT, which posits that self-efficacy serves as the core cognitive mechanism in the relationship between environmental support and career development. In the realm of Chinese engineering postgraduate education, supervisor support is associated with engineering self-efficacy, which in turn is associated with postgraduates’ identification with the engineer role. Specifically, the role of supervisor support in fostering engineering self-efficacy can be understood through the four pathways identified by social cognitive theory (Gladstone and Cimpian, 2021). First, supervisors’ academic guidance provides mastery experiences—opportunities for postgraduates to build confidence through successful task completion. Second, supervisors serve as role models, enabling vicarious learning. Third, supervisors’ encouragement and affirmation constitute verbal persuasion, which is associated with postgraduates’ capability beliefs. Fourth, supervisors’ emotional care may help regulate emotional arousal, potentially providing a stable psychological state for tackling challenges. These sources of self-efficacy are closely tied to supervisors’ supportive behaviors (Amador-Campos et al., 2023), showing how supervisor support is associated with engineering self-efficacy. Within the Chinese mentor-responsibility system and research group culture, the inclusive and collaborative team atmosphere fostered by supervisors is also associated with the development of postgraduates’ engineering self-efficacy.

In the process, engineering self-efficacy is associated with engineering identity through its relationship with behavioral engagement and identity internalization. Postgraduates with higher engineering self-efficacy are more inclined to actively participate in engineering practices and professional communities. Through sustained successful experiences and positive feedback, they gradually internalize the engineer role into their self-concept (Park et al., 2018). In this process, self-efficacy is associated with greater resilience in confronting technical challenges, as well as stronger identification with the value of the engineering profession and greater clarity about one’s professional positioning (Wei et al., 2025). In Chinese engineering education, which emphasizes industry-academia-research integration, postgraduates with strong engineering self-efficacy demonstrate greater clarity in academic exploration and career choices. This clarity is, in turn, associated with their engineering identity through industry practice and peer interactions. Consequently, engineering self-efficacy functions as a cognitive intermediary in the association between supervisor support and engineering identity, illustrating the link between external resources and the internalization of professional identities.

### 5.3 The moderating role of psychological capital

This study found that psychological capital moderates the relationship between supervisor support and engineering identity among engineering postgraduates. For postgraduates with higher levels of psychological capital, the positive association between supervisor support and engineering identity is stronger. This finding reveals the boundary-condition role of individual internal resources in the relationship between environmental factors and identity outcomes. Theoretically, postgraduates with higher psychological capital are more inclined to interpret supervisors’ academic guidance, emotional encouragement, and autonomy support as growth-promoting resources, and to proactively leverage such support, which is associated with a more stable engineering identity (Alismail et al., 2025). In contrast, postgraduates with low psychological capital exhibit greater passivity in perceiving and utilizing external support, which is associated with lower engineering identity. This pattern of findings aligns closely with PEIT: individual internal resources not only shape how individuals perceive and respond to the external environment but also influence when that environment is associated with positive outcomes. Within the Chinese mentor-responsibility system and research group culture, the moderating role of psychological capital takes on particular significance. Postgraduates with high psychological capital are better able to identify information and opportunities conducive to their professional growth from interactions with advisors, and hold stronger beliefs in overcoming difficulties and achieving career goals (Li et al., 2023). This positive psychological orientation is associated with deeper internalization of advisor guidance, a stronger sense of belonging to the engineering professional community, and higher future career pride (Hernandez et al., 2017). These findings suggest that psychological capital, as a developable positive psychological resource, offers an important intervention point for enhancing engineering identity among postgraduates.

Furthermore, the pattern of main effects in the moderation model warrants further discussion. Although the main effect of supervisor support was significant in the mediation model, it became non-significant in the moderation model—a pattern consistent with the significant interaction effect. This indicates that the relationship between supervisor support and engineering identity varies with the level of psychological capital. The significantly negative main effect of psychological capital may be explained by the possibility that individuals with high psychological capital hold higher expectations of environmental support; when supervisor support is at an average level, these unmet expectations may be temporarily and negatively associated with their engineering identity. However, the significant positive interaction term suggests that as supervisor support increases, the negative association of psychological capital may be gradually offset, potentially transforming into a reinforcing factor at high levels of supervisor support. This pattern of findings further empirically corroborates the core logic of PEIT.

### 5.4 Theoretical and practical contributions

This study makes a threefold theoretical contribution. First, we show that supervisor support is positively associated with engineering postgraduates’ engineering identity—a finding that situates supervisor support as a multidimensional contextual factor that relates to professional identity development. These findings add some empirical support for professional identity development literature. Second, by incorporating engineering self-efficacy and psychological capital, we reveal both mediating and moderating processes in the relationship between supervisor support and engineering identity. This not only deepens our understanding of the dynamic development process of engineering postgraduates’ identity, but also provides a new theoretical perspective on the boundary conditions and underlying mechanisms of this relationship. Third, based on the empirical data of Chinese engineering postgraduates, this study expands the scope of cross-cultural discussion on relevant topics. It adds important contextual data for understanding the general relation between supervisor support and professional identity construction globally.

The research offers practical suggestions for universities seeking to support engineering postgraduates’ identity. First, establish a multi-dimensional and collaborative supervisor support system. Universities should integrate the supportive supervisor–student relationships into the quality assurance framework of engineering postgraduate education. This can be achieved by creating clear guidelines for supervision behaviors, carrying out regular supervisor training programs, and setting up awards for outstanding supervisor teams. These initiatives are intended to facilitate coordinated support in the three dimensions of academic, emotional, and autonomy support. In guiding practice, tutors ought to provide research resources and clear academic guidance while also fostering empathetic communication and trust-based relationships. Additionally, supervisors should grant postgraduates reasonable autonomy in key processes, such as topic selection and experimental design, which can provide essential support for the establishment of their engineering identity. Second, universities should adopt a scaffolded approach for developing engineering self-efficacy. Universities ought to optimize their scientific research and training system by building a progressive task sequence—“foundational training → comprehensive practice → independent research”—designed to help postgraduates accumulate successful experience aligned with their developmental levels. They should develop a collection of excellent case and role model profiles to facilitate vicarious learning, which may help enhance postgraduates’ confidence in their engineering capabilities. Tutors should implement scaffolding strategies, dynamically adjusting the degree of intervention according to the ability level of postgraduates, gradually reducing intervention as structured support is internalized. Through specific, positive feedback, supervisors can help postgraduates attribute their successes to their own efforts, which may reinforce their sense of control over engineering tasks. Third, improve the mechanism of cultivating psychological capital. Universities can offer specialized programs, such as research resilience training and growth-mindset workshops. By integrating counseling services with academic support resources, universities can establish a development support center that conducts dynamic psychological assessments and provides tiered interventions. Supervisors should integrate psychological capital development into daily supervisor-student interactions. During critical phases, such as when postgraduates navigate research challenges, tutors can help students explore the path of success to cultivate hope. When postgraduates encounter experimental setbacks, supervisors can guide them toward positive attribution to cultivate optimism. When research projects face delays, supervisors can support them through emotional validation and meaning-making to enhance resilience.

## 6 Limitations and future study

Despite the substantial value and implications of this study, it has certain limitations. First, this study is limited by its cross-sectional design and the reliance on self-reported data collected at a single point in time. Therefore, the findings mainly reflect covariations and statistical associations, and it is not possible to determine their directionality or temporal sequence. The potential for common method bias is present. Future research may adopt longitudinal designs and use multi-source data, or cross-lagged designs, to better capture dynamic processes and to further examine possible directional relationships. Secondly, the sample was exclusively obtained from Chinese universities, hence constraining the generalizability of the findings to diverse cultural and educational environments. The relationship between supervisor support and engineering identity may be influenced by the unique Chinese tradition of mentorship, the policy environment for supervisor-student relationships, and societal expectations of engineering postgraduates. Future investigations could examine this study model across various cultural contexts to elucidate the generalizability of its application and its boundary requirements. Finally, our study examines the mediating role of engineering self-efficacy and the moderating effect of psychological capital, offering a singular viewpoint on the intricate interaction between supervisor support and the engineering identity of postgraduates in engineering. It is worth noting that social cognitive theory views professional identity as developing through the interaction of multiple personal and contextual factors (Eliot and Turns, 2011). Additional variables, such as learning engagement, school belonging, organizational climate, etc., may be incorporated to evaluate more complex mediation and moderation models (Jones et al., 2013; Sanchez et al., 2024; Bahnson et al., 2023).

## 7 Conclusion

The study examined the association between supervisor support and engineering identity, highlighting the mediating role of engineering self-efficacy and the moderating role of psychological capital. The main findings were consistent with previous studies in showing that supervisor support is positively associated with engineering identity (*β* = 0.270). The mediation model illustrated that engineering self-efficacy partially mediated the relationship between supervisor support and engineering identity. It was also shown that psychological capital acted as a significant moderator of the association between supervisor support and engineering identity. Conclusively, the positive association was stronger in postgraduates with high psychological capital. For engineering talent development, institutions should recognize the important role of supervisors in postgraduates’ identity development and enhance supervisor support through system training. Focus should also shift toward enhancing engineering postgraduates’ engineering self-efficacy and cultivating psychological capital, as these factors may strengthen the positive association between supervisor support and engineering identity, thereby supporting the quality of talent development among engineering postgraduates.

## Data Availability

The original contributions presented in the study are included in the article/supplementary material, further inquiries can be directed to the corresponding author.
